# Feasibility and Safety Study of Concomitant Left Bundle Branch Area Pacing and Atrioventricular Node Ablation with Same-Day Hospital Dismissal

**DOI:** 10.3390/jcm12227002

**Published:** 2023-11-09

**Authors:** Zhigang Liu, Xiaoke Liu

**Affiliations:** 1 Department of Cardiology, Ascension Borgess Hospital, Kalamazoo, MI 49048, USA; 2 Department of Cardiovascular Medicine, Mayo Clinic Health System, La Crosse, WI 54601, USA; 3 Department of Cardiovascular Medicine, Mayo Clinic, Rochester, MN 55901, USA

**Keywords:** atrial fibrillation, atrioventricular node ablation, left bundle branch area pacing, same-day dismissal, axillary vein access

## Abstract

Background: Left bundle branch area pacing (LBBAP) has rapidly emerged as a promising modality of physiologic pacing and has demonstrated excellent lead stability. In this retrospective study, we evaluate whether this pacing modality can allow concomitant atrioventricular node (AVN) ablation and same-day dismissal. Methods: Twenty-four consecutive patients (female 63%, male 37%) with an average age of 78 ± 5 years were admitted for pacemaker (75%)/defibrillator (25%) implantations and concomitant AVN ablation. Device implantation with LBBAP was performed first, followed by concomitant AVN ablation through left axillary vein access to allow for quicker post-procedure ambulation. The patients were discharged on the same day after satisfactory post-ambulation device checks. Results: LBBAP was successful in 22 patients (92% in total, 20 patients had an LBBP and two patients had a likely LBBP), followed by AVN ablation from left axillary vein access (21/24, 88%). All patients had successful post-op chest x-rays, post-ambulation device checks, and were discharged on the same day. After a mean follow up of three months, no major complications occurred, such as LBBA lead dislodgement requiring a lead revision. The LBBA lead pacing parameters immediately after implantation vs. three-month follow up were a capture threshold of 0.8 ± 0.3 V@0.4 ms vs. 0.6 ± 0.3 V@0.4 ms, sensing 9.9 ± 3.9 mV vs. 10.4 ± 4.1 mV, and impedance of 710 ± 216 ohm vs. 544 ± 110 ohm. The QRS duration before and after AVN ablation was 117 ± 32 ms vs. 123 ± 14 ms. Mean LVEF before and three months after the implantation was 44 ± 14% vs. 46 ± 12%. Conclusion: LBBA pacing not only offers physiologic pacing, but also allows for a concomitant AVN ablation approach from the left axillary vein and safe same-day hospital dismissal.

## 1. Introduction

The COVID-19 pandemic has dramatically affected the practice pattern in clinical cardiac electrophysiology. A wide variety of complex EP ablation and device implant procedures are currently performed with a same-day dismissal protocol in many institutions across the world [1,2]. This leads to decreased hospital stays with cost-saving implications, a potentially decreased risk of infection, and improved patient comfort and satisfaction without sacrifice in the clinical outcomes [1,2]. 

AVN ablation combined with pacemaker implantations has become a well-established, effective treatment strategy usually offered to patients with symptomatic AF with a rapid ventricular response (RVR) refractory to medical therapy, especially in the elderly population [3,4]. Patients in this category are usually nonresponsive to (or cannot tolerate) AVN-blocking agents, are of an advanced age or have significant comorbidities, and are not considered candidates for other more invasive curative procedures, such as catheter or surgical ablative therapies for AF [5]. One of the main concerns of the pace-and-ablate approach is that patients will become dependent on having a permanent pacemaker (PPM). Therefore, patients undergoing this treatment strategy typically have a PPM implanted first, followed by a waiting period (overnight to >30 days) to ensure lead stability and normal PPM function prior to AVN ablation.

More recently, LBBAP has rapidly emerged as a promising alternative to RV apex pacing, CS pacing, and even HIS bundle pacing, especially for those expected to have high RV pacing burdens [5,6,7]. LBBAP has the potential advantages of a stable lead position, a better pacing threshold, and sensing, when compared with conventional CS pacing and HIS pacing [8]. These advantages potentially make it feasible and safe to combine LBBAP and AVN ablation in one procedure and allow same-day hospital dismissal; although, larger clinical studies are needed to validate the preliminary data [9,10,11].

In this cohort study, we evaluate the feasibility and safety of concomitant LBBAP and AVN ablation through left axillary vein access with a same-day hospital dismissal after combining LBBAP and AVN ablation.

## 2. Methods

This was a retrospective data analysis performed at the Ascension Borgess Heart Institute in Michigan, USA. The study was reviewed and granted an exempt status by the Institutional Review Board at the Western Michigan University School of Medicine with IRB # WMed-2021-0836.

Twenty-four consecutive patients were included in this study for the pace-and-ablate strategy after a failed pharmacological AF rate control/rhythm control and AF ablation. The procedures were performed between June 2020 and April 2023. A total of 6 patients received cardiac resynchronization therapy–defibrillator (CRT-D) with LBBAP due to a severely reduced left ventricular ejection fraction (LVEF) of less than 35%. In addition to the LBBAP lead, either the RV defibrillation leads (as part of CRT-D) or conventional RV apical pacing leads (as part of CRT-P or potential backup leads) were inserted. None of the patients received a single-chamber PPM. All patients presented with longstanding chronic AF; as a result, no right atrial (RA) leads were inserted.

All 24 patients were on anticoagulants, either warfarin or direct oral anticoagulants (DOACs) for stroke prophylaxis prior to the procedure. Warfarin was continued before and after the procedure with INR goals of 2 to 3 regardless of the CHADS2-VAsc score. In patients with no prior history of TIA/stroke or a CHA2DS2-VASc score < 6, DOACs were typically held for 2 doses if they were dosed twice a day and 1 dose if dosed once a day. DOACS were otherwise only held on the day of the procedure. Depending on whether there was evidence of significant bleeding intra-operatively, DOACs were either resumed on the same day for high-risk patients or the next day for patients without high-risk features (prior history of TIA/stroke or a CHA2DS2-VASc score of 6 or higher). If there was evidence of active bleeding post-operatively (growing hematoma), DOACS or Warfarin was held until there was evidence of hemostasis based on a clinical assessment.

The patients were brought to the EP laboratory and underwent a standard left precordial preparation and draping.

Venous access was achieved by a left axillary vein puncture guided by fluoroscopy. The right ventricular lead (either an ICD lead or regular pacing lead) was first implanted using the standard technique in the right ventricular apex under fluoroscopy guidance. After the confirmation of adequate pacing and sensing parameters, the lead was then secured to the underlying tissue and connected to the RV port of the CRT-D or dual-chamber PPM.

**Figure 1:**
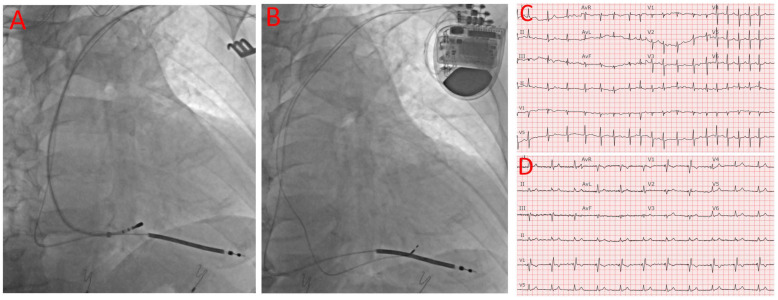
Device implantation, AV node ablation, baseline ECG, and post-procedural ECG. (**A**) ICD RV lead position and mapping of AV node. (**B**) Final positions of ICD RV and LBBAP leads. (**C**) Baseline ECG showing AF with RVR and narrow QRS. (**D**) Post-procedural ECK showing LBBA pacing with narrow QRS.

A deflectable EP mapping/ablation catheter was inserted to map the His bundle signal, and the His position was then recorded by cine imaging to facilitate AV node ablation and the subsequent implantation of the LBBAP lead. Figure 1A shows the mapping of the His position after the RV lead insertion. At this time, the device was programmed to VVI with a lower rate of 40 for backup pacing, and the ablation catheter was inserted to ablate the AV node. A standard CS delivery sheath was used to facilitate the catheter reach and to maintain stability, and a deflectable sheath would have been used if the ablation remained challenging with the standard CS delivery sheath. After achieving adequate, stable contact with the compact AV node, ablation was then initiated, typically at 50 W with a temperature upper limit of 65 degrees Celsius. After the confirmation of complete heart block, the pacing rate was typically changed to VVI 90 for the rest of the procedure. In cases where the AVN could not be successfully ablated from the superior approach, the conventional femoral approach was used after the completion of the device implant. Vascular closure devices were used to help achieve hemostasis and promote early mobilization after the procedure.

The LBBAP lead implantation was then performed using the same technique as previously reported [12]. Briefly, a C315His delivery sheath (Medtronic, Minneapolis, MN, USA) was advanced into the RV over a standard J-wire. The 3830 lead (Medtronic, Minneapolis, MN, USA) was then advanced through the delivery sheath to the right ventricular septum about 1–2 cm distal to the His bundle region. Once the lead was pointed perpendicular to the septum, manual rotations were applied with forward pressure. Pacing from the 3830 lead was then performed in a unipolar fashion after every 1–2 rotations until the paced morphology demonstrated a narrow RBBB morphology indicating the capture of the LBBA. In addition to the RBBB pattern in V1, the LBBAP capture was confirmed by one of the following criteria: (1) an output-dependent transition in QRS morphology, either from non-selective left bundle branch pacing (LBBP) to selective left bundle branch pacing or from non-selective LBBP to left ventricular septal pacing (LVSP) at decremental voltage output pacing; (2) short- and stable-paced stimulus to V6 RWPT <75 ms in narrow QRS and <80 ms in an LBBB/interventricular conduction delay (IVCD); (3) V6-V1 interpeak interval > 44 ms, and (4) delay of the left bundle branch potential to V6RWPT (time to peak R wave in V6) in an intrinsic rhythm equal to the pacing stimulus of V6RWPT [13]. Based on these proposed criteria, 20 out of the 24 patients had a definite LBBP (84%), 2 out of the 24 patients likely had a LBBP (8%), 1 patient had nonselective HIS bundle pacing (4%), and 1 patient had deep septal pacing (4%). Figure 1B shows both RV and LBBAP leads are inserted. Cases that did not meet the abovementioned criteria but still had a RBBB pattern and relatively narrow QRS (<120 ms with a baseline normal QRS or <130 ms with a baseline wide QRS) were considered to be deep septal pacing.

After the confirmation of adequate pacing and sensing parameters, the LBBA pacing lead was then secured to the suture sleeve, which was then secured to the underlying tissue with a non-absorbable suture. The 3830 lead was then connected to the LV port of the CRT or RA port of the dual-chamber PPM. The LBBA pacing was set to 80 ms ahead of the RV apex pacing. For patients receiving a dual-chamber device with an RV backup pacing lead, the device was programmed to AAIR with VVIR backup pacing and the safety pacing mechanism was turned off. The average fluoroscopic time of the procedure was 10.2 ± 4.1 min.

The patients were then transported to the cardiac recovery unit after the procedure, where they were observed for 3 to 4 h. A chest X-ray was performed to document the lead placement and rule out acute pneumothorax. Patients were allowed to ambulate after recovery from sedation without prolonged bedrest because of the absence of femoral venous access for the AVN ablation, or in rare cases that required the femoral approach, vascular closure devices were used to minimize the bedrest time. The devices were interrogated after ambulation and before discharge. All 24 patients were discharged on the same day of the procedure.

The patients were followed up in the EP clinic after about one week for the incision inspection and device interrogation. Further regular follow ups were schedule three and six months after the procedure when the device parameters were checked and documented, in addition to an EKG and echocardiogram, which were performed approximately three months after the procedure.

All the data were expressed as the mean ± standard deviation. The student t-test was used to evaluate the statistical significance. A *p*-value of 0.05 or lower was considered statistically significant.

## 3. Results

LBBAP was successful for 22 out of the 24 patients (92% in total, 20 patients had an LBBP and two patients likely had an LBBP), followed by AVN ablation from left axillary vein access in all but three patients who required the standard right femoral approach. As shown in Table 1 for the baseline characteristics, the average age of our patients was 78 ± 5 years old. The majority were female patients (63%). All the patients presented with longstanding persistent AF with multiple comorbidities and multiple medications for either rate control or rhythm control. No RA lead was implanted considering the unlikelihood of a spontaneous conversion back to a sinus rhythm. The average pre-procedure LVEF was 44 ± 14%. Six out of the 24 patients (25%) had severely reduced LVEF values of less than 35%, and these patients underwent CRT-D implantations with an LBBAP. LBBAP instead of CS pacing was chosen for the patients with an EF < 35% because these patients did not have baseline left bundle branch blocks and CS pacing was previously not shown to improve the outcomes in patients with a narrow QRS [14,15]. Four out of the 24 patients (17%) presented with an LVEF greater than 35% but less than 50%, and these patients received an RV pacing lead, in addition to LBBAP, for cardiac resynchronization therapy. The rest of the patients (14 patients, 58%) also received RV pacing leads as backup leads in case of LBBAP failure and the potential future indication of cardiac resynchronization therapy. Overall, no significant changes in QRS duration were observed before and after the ablation, with the before-and-after-procedure QRS durations being 117 ± 32 ms vs. 123 ± 14 ms. A further analysis showed a significant reduction in a QRS duration > 20 ms in five patients (21%) who had a baseline QRS duration > 140 ms, while nine patients (37.5%) demonstrated a slightly increased QRS duration >10 ms, with a baseline QRS duration < 100 ms. Figure 1C,D shows the ECG before and after the procedure.

No major complications were noted immediately after the procedure, except that one patient developed a small pocket hematoma, but no evacuation was needed, as shown in Table 2. The patient was observed in a post-surgery observation unit for 3 to 4 h before the same-day discharge. A chest X-ray was performed for all cases; no acute pneumothorax was seen and no lead macro-dislodgement was detected either. All patients were on anticoagulants, either a direct oral anticoagulant (DOAC) or warfarin, which were restarted after the procedure. The patients ambulated as soon as they recovered from the procedural sedation. Devices were interrogated after patient ambulation to confirm the lead stability. No significant changes in the lead parameters, including RV leads and LBBAP leads, were observed prior to the dismissal.

**Figure 2:**
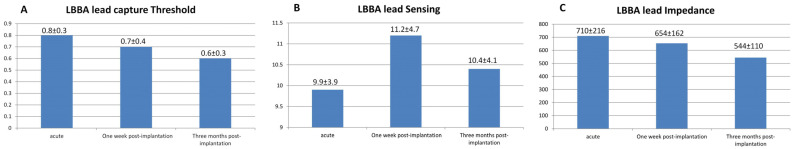
Device parameter measurements during implantation: one-week and three-month follow ups (mean ± standard deviation). (**A**) LBBA lead capture threshold, unit in V@0.4 ms. (**B**) LBBA lead sensing, unit in mV. (**C**) LBBA lead impedance, unit in Ohm.

After a mean follow up of 3 months, all patients remained in complete heart block with no evidence of AVN recovery. As demonstrated in Table 3 and Figure 2, the LBBAP lead parameters are stable. No significant changes in the LBBAP lead capture threshold were noted after three months of having the implantation (0.8 ± 0.3 V@0.4 ms vs. 0.6 ± 0.3 V@0.4 ms). There was a slight increase in the LBBAP lead sensing one week after the implantation; then, the sensing trended downward again (sensing 9.9 ± 3.9 mV vs. 10.4 ± 4.1 mV). The LBBAP lead impendence decreased steadily (710 ± 216 ohm vs. 544 ± 110 ohm). No data are presented beyond three months after the ablation, but our follow up shows that the impendence stabilizes three months after the implantation without a further trend of decreasing.

As mentioned above, an echocardiogram was repeated three months after the procedure. Overall, no significant LVEF change was observed before or after the procedure (44 ± 14% vs. 46 ± 12% (*p* > 0.05)).

Despite the lack of significant overall LVEF changes before and after the procedure, all 24 patients experience clinical improvements in symptoms related to AF and reported an improved overall quality of life after the procedure.

## 4. Discussion

Concerns about health-care-associated infections and financial cost secondary to extended hospital stays have led to more and more complex EP procedures being performed using a same-day dismissal protocol in the USA, especially since the onset of the COVID-19 pandemic. Multiple studies have confirmed the safety of same-day dismissals for device implantations as well as complex ablation procedures, such as atrial fibrillation ablation [16,17]. Nevertheless, because of the concerns about lead dislodgement and pacing dependency, traditional PPM implantations and AVN ablation procedures are still routinely performed at separate times and typically require two separate hospital admissions and at least one overnight stay in the hospital. In this study, we provided initial feasibility and safety data that suggested this type of procedure may also be performed using a same-day dismissal protocol. Multiple additional procedures were implemented clinically to ensure safety and to facilitate same-day dismissals. First, ventricular pacing was performed through an LBBAP in patients who only required RV pacing and in patients who qualified for a CRT. Compared to the conventional RV apical lead that is fixated superficially in the myocardial tissue, the LBBAP lead is implanted deep into the septal myocardium, which confers superb lead stability and excellent pacing parameters, as shown in this and multiple other studies. Moreover, compared to conventional CRT with CS pacing and other modalities of conduction system pacing, such as His bundle pacing, LBBA pacing has the potential advantages of a stable lead position, a better pacing threshold, and sensing [8]. Compared to the His bundle pacing lead, LBBAP and AVN ablation are associated with a higher success rate and fewer lead-related complications [18].

The second safety measure we employed in this study was the implantation of a backup RV pacing lead. This was made possible as all patients in the study had persistent atrial fibrillations and either the atrial port (for dual-chamber PPM) or the LV port (for CRT device) could be used for the LBBAP while the standard RV port was available for the backup RV pacing lead. In the unlikely event of acute LBBAP lead dislodgement, the backup RV lead can serve as the safety net to provide pacing support and allow time for lead revision.

The third new procedure we adopted for this study was the approach of AVN ablation through left axillary vein access, which was later used as the access for the LBBAP lead. AVN ablation using the axillary vein has been recently studied and appears to have multiple inherent advantages compared to the conventional right femoral approach [16,17]. This approach is even more relevant in cases involving an LBBAP because the His bundle region is typically already mapped prior to the LBBAP lead implant, and the venous access for AVN ablation is also already established. The only additional step required is the insertion of an ablation catheter (frequently over a long sheath to enhance stability) to ablate the AVN, guided by the His bundle position already recorded as part of the LBBAP procedure. Only one patient in this series required right femoral access. In this case, a vascular closure device was used to minimize the bedrest time. By eliminating (or minimizing) femoral access, patients are able to ambulate sooner, which translates to better patient comfort, quicker recovery, and typically facilitates a same-day dismissal.

In addition to the abovementioned measures, we added another protocol of performing a post-ambulatory device check after an immediate post-op chest X-ray confirming the lead position and adequate slack. Patients were only discharged if all these checks were satisfactory.

Finally, it should be noted that the majority of the patients in this study demonstrated a reliable escape rhythm (16/24) during the office follow up. This may be related to our intentional use of a relatively small-tip catheter (≤5 mm) and attempt to ablate the AV node as proximately as possible to potentially increase the chance of a post-ablation junctional escape rhythm.

Interestingly, a dominant percentage of the patients were female (63%) in this study. This gender bias might reflect the findings published by Linde et al. that female patients are less likely to undergo cardioversion and AF ablation, but more likely to undergo AVN ablation therapy than male patients, even though female patients have more severe AF symptoms [19].

## 5. Limitation

The small sample size in this study limited the power of this clinical study. In addition, this was a retrospective and observational study. However, our conclusions are consistent with larger studies demonstrating the high stability and low complication rate of the LBBAP lead [20,21]. A large-scale randomized clinical study is needed to further validate the preliminary conclusions of this study.

## 6. Conclusions

In this single-center pilot study, we provide initial evidence that it is feasible and safe to perform concomitant LBBAP and AVN ablation through the left axillary vein with same-day hospital dismissals.

## Figures and Tables

**Table 1:** Patient baseline characteristics.

**Baseline Characteristics (N = 24)**	
Age ± SD (year old)	78 ± 5
**Gender**	
Male (%)	9 (37%)
Female (%)	15 (63%)
**AF type**	
Persistent (%)	24 (100%)
Paroxysmal (%)	0 (0%)
**Type of device implanted**	
CRT-D (%)	6 (25%)
Dual-chamber PPM (%)	18 (75%)
**LVEF**	44 ± 14
**Pre-implantation QRS duration (ms)**	117 ± 32
**Comorbidities**	
Diabetes (%)	10 (42%)
Hypertension	19 (79%)
Coronary artery disease	11 (46%)
Chronic obstructive lung disease	4 (17%)
Cerebrovascular accident	3 (13%)
**Medication**	
Beta-blocker	20 (83%)
Non-dihydropyridine calcium channel blocker	10 (42%)
Digoxin	5 (21%)
Class I C antiarrhythmic drug	3 (13%)
Class III antiarrhythmic drug	9 (38%)

**Table 2:** Procedure-related complications.

Complication (N = 24, Duration of Follow up: 90 Days)	
**Pocket hematoma, no intervention needed**	1
Pocket hematoma requiring intervention	0
Pneumothorax	0
Cardiac perforation (including tamponade)	0
Lead dislodgement requiring revision	0
Infection	0

**Table 3:** Post-implantation device parameters: QRS width and LVEF.

Post-Implantation Device Parameters: QRS Width and LVEF (N = 24)	
**LBBA lead Capture Threshold (V@0.4 ms)**	
Acute	0.8 ± 0.3
One week post-implantation	0.7 ± 0.4
Three months post-implantation	0.6 ± 0.3
**LBBA Lead Sensing (mV)**	
Acute	9.9 ± 3.9
One week post-implantation	11.2 ± 4.7
Three months post-implantation	10.4 ± 4.1
**LBBA Lead Impedance (Ohm)**	
Acute	710 ± 216
One week post-implantation	654 ± 162
Three months post-implantation	544 ± 110
**Post-implantation QRS duration (ms)**	123 ± 14
**Post-implantation LVEF (%)**	46 ± 12

## Data Availability

Data are contained within the article.
